# Chemorheological Monitoring of Cross-Linking in Slide-ring Gels Derived From *α*-cyclodextrin Polyrotaxanes

**DOI:** 10.3389/fchem.2022.923775

**Published:** 2022-07-14

**Authors:** Karan Dikshit, Carson J. Bruns

**Affiliations:** ^1^ Materials Science and Engineering Program, University of Colorado Boulder, Boulder, CO, United States; ^2^ Paul M. Rady Mechanical Engineering Department, University of Colorado Boulder, Boulder, CO, United States; ^3^ ATLAS Institute, University of Colorado Boulder, Boulder, CO, United States

**Keywords:** chemorheology, rheology, cyclodextrins, organogels, slide-ring gels, polyrotaxane, viscoelasticity

## Abstract

Despite hundreds of studies involving slide-ring gels derived from cyclodextrin (CD)-based polyrotaxanes (PRs), their covalent cross-linking kinetics are not well characterized. We employ chemorheology as a tool to measure the gelation kinetics of a model slide-ring organogel derived from *α*-cyclodextrin/poly (ethylene glycol) PRs cross-linked with hexamethylenediisocyanate (HMDI) in DMSO. The viscoelastic properties of the gels were monitored *in situ* by small-amplitude oscillatory shear (SAOS) rheology, enabling us to estimate the activation barrier and rate law for cross-linking while mapping experimental parameters to kinetics and mechanical properties. Gelation time, gel point, and final gel elasticity depend on cross-linker concentration, but polyrotaxane concentration only affects gelation time and elasticity (not gel point), while temperature only affects gelation time and gel point (not final elasticity). These measurements facilitate the rational design of slide-ring networks by simple parameter selection (temperature, cross-linker concentration, PR concentration, reaction time).

## Introduction

Main-chain polyrotaxanes are mechanically bonded macromolecules with sliding bead-on-string structures which, when appropriately cross-linked, give rise to slide-ring networks Okumura and Ito (2001); Mayumi et al. (2015) having remarkable stress-dissipative properties. Mayumi and Ito (2010) The most highly researched slide-ring networks are derived from the poly (ethylene glycol) (PEG)/*α*-cyclodextrin (*α*-CD) polyrotaxanes first introduced by Harada, Harada et al. (1992) due in large part to their favorable cost, biocompatibility, and accessibility. Araki and Ito (2007) These networks can exhibit high softness, Kato et al. (2013) extensibility, Bin Imran et al. (2014); Kato et al. (2015b); Jiang et al. (2018) and fracture toughness, Liu et al. (2017, 2019) leading to varied applications in technologies such as cell-growth substrates Hyodo et al. (2019); Rajendran et al. (2019), dental materials Matsunaga et al. (2020), piezoelectric sensorsSeo et al. (2021), scratch-resistant coatings, Noda et al. (2014); Seo et al. (2019) and battery electrode binders. Choi et al. (2017); Cho et al. (2019); Yoo et al. (2019) Despite the growing reach of slide-ring gels, Hart et al. (2021) reports on their kinetics are limited mainly to gel degradation Kang et al. (2021) or physical gels formed by hydrogen-bond-driven *α*-CD micro-crystallization. Travelet et al. (2009, 2010a,b).

In the course of our own slide-ring experiments, Dikshit and Bruns (2021) we sought to characterize the kinetics of chemically cross-linked polyrotaxane gels in order to calibrate our reaction times. Here we chemically cross-link *α*-CD PRs and use SAOS rheological time sweeps to characterize the gelation process *in situ* by observing the temporal evolution of the viscoelastic moduli. We show how the kinetics and mechanics of a model slide-ring gel depend on temperature, cross-linker concentration, PR concentration, and reaction time. This information will facilitate the selection and fine-tuning of desired network structure and properties.

## Results and Discussion

**SCHEME 1:**
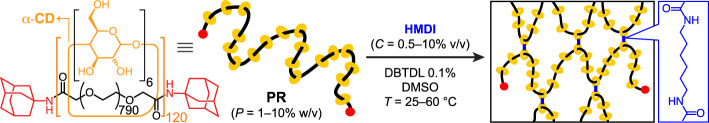
Synthesis of slide-ring gels from *α*-CD/PEG polyrotaxane (PR) and cross-linker HMDI.

We chose to measure the chemical gelation kinetics by chemorheology Halley and Mackay (1996) to serve our interests in gel mechanics. The PR physical gelation studies Travelet et al. (2009, 2010a,b) have validated a rheological approach to measuring gelation kinetics in PR systems. Among a variety of hydroxyl-reactive cross-linkers, Karino et al. (2006); Samitsu et al. (2006); Shinohara et al. (2006); Ito (2007) HMDI has been reacted with CD-based PRs to give slide-ring gels, Kato et al. (2013) adhesives, Dikshit and Bruns (2021) elastomers, Minato et al. (2017) nanocomposites, Iida et al. (2021) polymer electrodes, Lin et al. (2018) and piezoelectric sensors. Seo et al. (2021) In our model system, we employ HMDI in DMSO with 0.1% v/v dibutyltin dilaurate (DBTDL) to cross-link (Scheme 1) the *α*-CD rings of an adamantamine-capped Araki et al. (2005) polyrotaxane (PR) based on a PEG backbone of molecular weight (MW) 35,000 Da and an inclusion ratio (proportion of threaded diethylene glycol units in the chain) of ∼30%, corresponding to ∼120 *α*-CD rings per chain based on NMR signal integration. The number-average molecular weight of 152 kDa obtained using NMR analysis (Supplementary Figure S1) is comparable to that estimated by gel permeation chromatography (138 kDa, Supplementary Figure S2). Specimen labels of the form *P*_*P*_ *C*_*C*_ *T*_*T*_ differentiate the samples based on PR concentration (*P*_*P*_), HMDI concentration (*C*_*C*_) and temperature (*T*_*T*_). By monitoring the isothermal evolution of viscoelastic moduli in SAOS, we describe how these parameters impact gelation kinetics and mechanical properties. Other gelation parameters, such as sample volume (0.5 ml) and reaction time (18 h), were held constant across all experiments.

**FIGURE 1:**
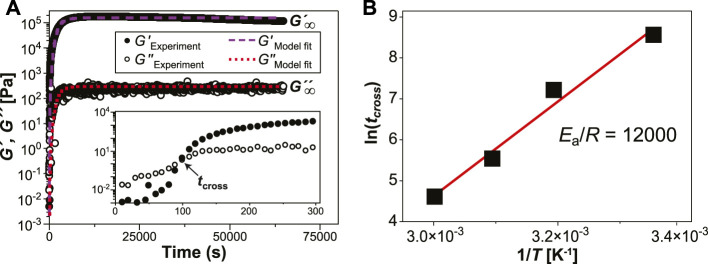
**(A)** Chemorheological time sweep of sample *P*_*10*_ *C*_*5*_ *T*_*60*_ over 68,400 s (18 h) show the plateau moduli (*G′,G″*) when gelation is complete. Inset magnifies the gel point at crossover time (*t*_*cross*_) in the first 300 s. **(B)** Activation energy of gelation. An Arrhenius plot of *t*_*cross*_ against 1/*T* gives an activation energy of 96 kJ/mol for the cross-linking reaction.

Figure 1A shows an example of the evolution of storage moduli (*G′*) and loss moduli (*G″*) in specimen *P*_*10*_ *C*_*5*_ *T*_*60*_ ([PR] = 10% w/v [HMDI] = 5% v/v, *T* = 60°C) over 18 h. The inset magnifies the data at the gel point, where *G’* and *G”* intersect at crossover time *t*_*cross*_. We performed the analogous sweeps while varying PR concentration from 1 to 10% w/v (Supplementary Figure S3, [HMDI] = 5% v/v, *T* = 60°C), HMDI concentration from 0.5 to 10% v/v (Supplementary Figure S4, [PR] = 10% w/v, *T* = 25°C), and temperature from 25 to 60°C (Supplementary Figure S5, [PR] = 10% v/v, [HMDI] = 5% w/v). A thin layer of heavy mineral oil is maintained around the sample throughout the *in situ* gelation to prevent drying. Without the ring of mineral oil, an 18-h time sweep of sample *P*_*10*_ *C*_*0*_ *T*_*25*_ (no cross-linker) exhibits an increase in viscoelastic moduli and gel point due to physical gelation associated with sample drying; in the presence of mineral oil the same solution does not dry and the moduli remain unchanged over the 18-h interval (Supplementary Figure S6). Isothermal time sweeps have been used to capture time-resolved viscoelasticity data in other cross-linking reactions, Chambon and Winter (1987); Adrus and Ulbricht (2013); Vo et al. (2020) where the evolution of *G′* and *G″* can be linked to specific processes in the sample. *G′* and *G″* are equal at the gel point, above which the material attains enough elastically effective cross-links to exhibit solid-like behavior. Vlassopoulos et al. (1998) The Arrhenius plot (Figure 1B) of ln (*t*_*cross*_) vs inverse temperature (1/*T*) was used to estimate Yang et al. (2015) a gelation activation energy of 96 kJ/mol. Since this activation energy exceeds that of small-molecule isocyanate-hydroxyl coupling, Cheikh et al. (2019) we infer that autoretardation due to cross-linker immobilization on the PR and nearest neighbor effects Alfrey and Lloyd (1963) are involved. This finding also supports the presumed mechanism of gelation driven by urethane formation between HMDI and *α*-CD, since the physical gelation process driven by hydrogen bonding in *α*-CD PRs has negative activation energy. Travelet et al. (2009) Further information can be extracted Sun Han Chang et al. (2019) from the time sweeps by fitting the data to the modified Hill Eq (1):

$$G^{\prime }\left(t\right)=G_{\infty }^{\prime }\frac{t^{\alpha }}{t^{\alpha }+\theta^{\alpha }}$$ (1)

where *θ* is gelation halftime, $G_{\infty }^{\prime }$ is the plateau modulus and *α* is an exponential fitting parameter. Adibnia and Hill (2016) The modified Hill equation is a kinetic equation adapted to understand systems that evolve with time, often used to evaluate dose response curves in pharmacology Giraldo et al. (2002) and in supramolecular chemistry. Gorteau et al. (2005); Benedini et al. (2021) The fitting parameters obtained from the modified Hill equation shed light on how cross-links form over time in the gel. Junior et al. (2019); Kodavaty et al. (2020) $G_{\infty }^{\prime }$ captures the stiffness of the final gel. *θ* reflects the speed of the reaction after *t*_cross_. These terms can be used to calculate (Table 1) the rate of production of elastically effective cross-links ( ${\dot{n}}_{\theta }$ ) at gelation halftime according to Eq (2).

$${\dot{n}}_{\theta }=\frac{\alpha G_{\infty }^{\prime }}{4\theta RT}$$ (2)

**TABLE 1:** Fitting parameters using modified Hill equation.

Specimen Name	*θ* [s]	G’_*∞*_[kPa]	*α*	P [Pa.s^−1^]
*P*_*1*_ *C*_*5*_ *T*_*60*_	6.49 × 10^4^	10.6	2.47	1.14
*P*_*2*_ *C*_*5*_ *T*_*60*_	4.17 × 10^4^	15.2	1.86	0.169
*P*_*5*_ *C*_*5*_ *T*_*60*_	1.94 × 10^4^	71.5	1.24	0.101
*P*_*10*_ *C*_*0.5*_ *T*_*25*_	6.02 × 10^4^	1.30	9.13	0.049
*P*_*10*_ *C*_*1*_ *T*_*25*_	5.67 × 10^4^	4.74	5.42	0.113
*P*_*10*_ *C*_*1*_ *T*_*50*_	2.79 × 10^3^	4.63	3.05	1.26
*P*_*10*_ *C*_*1*_ *T*_*60*_	1.43 × 10^3^	4.92	3.25	2.79
*P*_*10*_ *C*_*5*_ *T*_*25*_	5.18 × 10^4^	130	2.83	1.78
*P*_*10*_ *C*_*10*_ *T*_*25*_	1.72 × 10^4^	562	2.72	22.2
*P*_*10*_ *C*_*5*_ *T*_*40*_	2.90 × 10^4^	100	2.19	1.88
*P*_*10*_ *C*_*5*_ *T*_*50*_	1.06 × 10^4^	105	1.60	3.97
*P*_*10*_ *C*_*5*_ *T*_*60*_	2.88 × 10^3^	159	3.18	44.1

**FIGURE 2:**
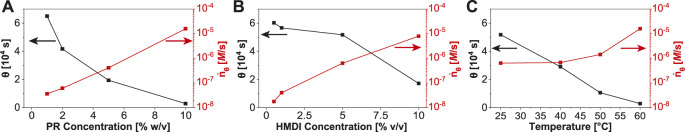
Tracking gelation as a function of reaction conditions. Gelation halftime (*θ*) and rate of production of elastically effective crosslinks ( ${\dot{n}}_{\theta }$ ) against **(A)** polyrotaxane concentration (60°C, 5% HMDI) **(B)** cross-linker concentration (25°C, 10% PR) and **(C)** temperature (5% HMDI, 10% PR).

The fitted data show that gelation can be accelerated by increasing PR concentration (Figure 2A), HMDI concentration (Figure 2B), or temperature (Figure 2C). The rate ${\dot{n}}_{\theta }$ increases by almost three orders of magnitude as the concentration of either PR or HMDI increases from 1 to 10%. We do not exceed 10% v/v for HMDI concentration because the pulley effect is suppressed Fleury et al. (2007) with increasing cross-link density, while 10% w/v is approaching the solubility limit of PR in DMSO. We could not obtain gels reliably below 1% PR or 0.5% HMDI concentrations. The rate ${\dot{n}}_{\theta }$ is insensitive to temperature over the range of 25–50°C, but increases by an order of magnitude between 50 and 60°C. The rate of crosslink production shows a second-order dependence with respect to the cross-linker concentration (Supplementary Figure S7), while it is 2.5 order with respect to PR concentration (Supplementary Figure S8). Using the empirical data (Table 1; Figure 2), the rate law for the production of elastically effective cross-links is determined to be:

$${\dot{n}}_{\theta }=\left(1.9\times 10^{4}M^{-3.5}s^{-1}\right){\left[HMDI\right]}^{2}{\left[PR\right]}^{2.5}$$

**FIGURE 3:**
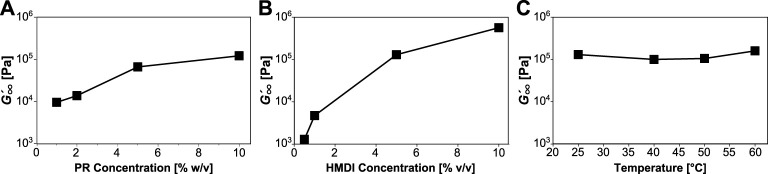
Post-gelation mechanics of slide-ring gels. Plateau modulus $\left(G_{\infty }^{\prime }\right)$ , which serves as a proxy for mechanical properties after completion of gelation, is plotted against **(A)** PR concentration **(B)** cross-linker concentration and **(C)** temperature. Concentration of HMDI, which dictates the formation of elastically effective crosslinks, has a significantly higher impact on $G_{\infty }^{\prime }$ than temperature and PR concentration.

The plateau modulus, $G_{\infty }^{\prime }$ , reflects the final count of cross-links when gelation is complete. As shown in Figure 3, $G_{\infty }^{\prime }$ is sensitive to PR concentration (Figure 3A) and highly sensitive to HMDI concentration (Figure 3B), but insensitive to temperature (Figure 3C). The plateau modulus increases by almost three orders of magnitude as [HMDI] increases from 0.5 to 10% v/v, while it increases approximately one order of magnitude as [PR] increases from 1 to 10% w/v. The insensitivity of $G_{\infty }^{\prime }$ to temperature, also observed in the case of sample *P*_*10*_ *C*_*1*_ *T*_*x*_ (*x* = 25, 50, or 60°C, Supplementary Figure S9), suggests that the final network structure is similar when [PR] and [HMDI] are held constant, given sufficient reaction time. This observation further implies that the solution-state structure of PR does not vary between 25 and 60°C, and that the cross-linker is consumed completely in all the experiments. We confirmed that HMDI is consumed completely over the 18-h interval by monitoring the gelation of *P*_*10*_ *C*_*5*_ *T*_*25*_ by *in situ* FTIR spectroscopy. The absorption band at 2,260 cm^−1^ attributable to the isocyanate group disappears completely as it is replaced by signals corresponding to the NH and C=O groups of the urethane cross-links (Supplementary Figure S10).

**FIGURE 4:**
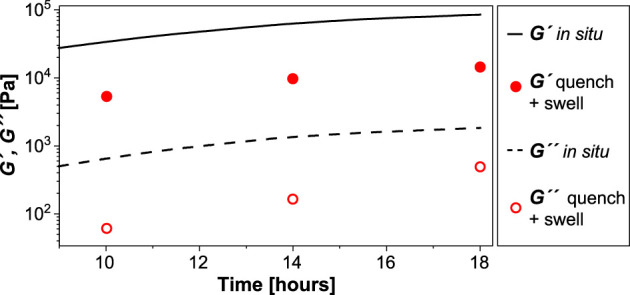
Controlling gelation and polyrotaxane mechanics. Storage and loss moduli values (circular symbols) of gels terminated at 10 h, 14 h, and 18 h are compared with the time sweep data of P_10_C_5_T_25_. The swelling ratios (by weight) of the organogel are mentioned at the specific time points in the gelation process.

We leveraged our time-resolved viscoelasticity data to demonstrate a novel way to tune slide-ring gel softness based on reaction time. We hypothesized that quenching the cross-linking reaction prior to completion would result in softer final gels with moduli that are predictably lower, proportional to the *in situ* viscoelastic moduli at quench time. Samples of *P*_*10*_ *C*_*5*_ *T*_*25*_ in glass scintillation vials were quenched at time points of 10, 14, and 18 h by the addition of excess methanol, followed by vacuum drying overnight at 70°C. The quenched gels were swollen in DMSO for 24 h before carrying out frequency sweeps (0.1–100 rad/s, strain 1%, Supplementary Figure S11). The red data points representing these gels, softened due to swelling, Subramani et al. (2020) are superimposed on the *in situ* sweep of *P*_*10*_ *C*_*5*_ *T*_*25*_ in Figure 4, showing uniformly lower values. The storage moduli of the swollen quenched gels increase from 5.3 kPa (10 h) to 14.4 kPa (18 h), while the corresponding loss moduli increase from 61 to 491 Pa, respectively. The ratio of *G′*_*18h*_:*G′*_*10h*_ is 2.7 *in situ* and 2.5 after quenching and swelling. Likewise, *G′*_*14h*_:*G′*_*10h*_ is 1.8 *in situ* and 1.9 after quenching and swelling. The nearly matching ratios confirm that the chemorheological sweeps can be used to predict and select desired modulus values (below the maximum) by quenching at an appropriate time in the evolution of the cross-linking reaction. The swelling ratio of the quenched gel decreases from 16 at 10 h to eight at 18 h (Supplementary Table S1), confirming the higher cross-link density of the latter sample. This quenching procedure provides an additional handle to fine-tune gel stiffness beyond HMDI concentration, which is a more course-tuning factor. Since it is performed at room temperature, it also reduces equipment and labor requirements.

## Conclusion

In summary, chemorheological characterization of polyrotaxane gelation kinetics provides insight on the evolution of gel elasticity and its dependence on polymer and cross-linker concentration, as well as temperature. Our kinetics data confirm that the mechanism of polyrotaxane gelation is driven by the formation of chemical cross-links and may also help researchers calibrate their gelation conditions to achieve desired gel structure and properties. The reaction is, unsurprisingly, most sensitive to cross-linker concentration [HMDI], which influences the gelation time, gel point, and final elasticity. PR concentration impacts the reaction rate more strongly, but the final gel elasticity is less affected, while the gel point is almost unchanged at all PR concentrations. The reaction temperature does not affect the final gel mechanics, but the total reaction time is reduced from 18 h at room temperature to less than 1 h at 60°C, information which is of practical consequence for preparing these gels. We also showed that the gelation may be arrested mid-reaction by quenching, and the elasticity of the resulting swollen gels are proportional to those made *in situ*. In addition to the experimental parameters probed here, other work has shown that factors such as PR molecular weight, Ohmori et al. (2016) inclusion ratio, Kato et al. (2015b); Jiang et al. (2018) ring size, Kato et al. (2015a) functional groups, Bin Imran et al. (2014); Liu et al. (2021) and type of cross linker Kato et al. (2019); Liu et al. (2021) also play major roles in the mechanical properties of slide-ring gels. Our chemorheological data add to this body of knowledge and enable judicious selection of reactant concentrations, temperature, and reaction time to control the viscoelastic properties of slide-ring gels over a wide range of modulus values between 1 kPa and 1 MPa.

## Data Availability

The original contributions presented in the study are included in the article/Supplementary Material. Further inquiries can be directed to the corresponding author.

## References

[B1] Adibnia V. Hill R. J. (2016). Universal Aspects of Hydrogel Gelation Kinetics, Percolation and Viscoelasticity from Pa-Hydrogel Rheology. J. Rheol. 60, 541–548. 10.1122/1.4948428

[B2] Adrus N. Ulbricht M. (2013). Rheological Studies on Pnipaam Hydrogel Synthesis via *In Situ* Polymerization and on Resulting Viscoelastic Properties. React. Funct. Polym. 73, 141–148. 10.1016/j.reactfunctpolym.2012.08.015

[B3] Alfrey T. Jr Lloyd W. G. (1963). Kinetics of High‐Polymer Reactions: Effects of Neighboring Groups. J. Chem. Phys. 38, 318–321. 10.1063/1.1733659

[B4] Araki J. Ito K. (2007). Recent Advances in the Preparation of Cyclodextrin-Based Polyrotaxanes and Their Applications to Soft Materials. Soft Matter 3, 1456–1473. 10.1039/b705688e 32900100

[B5] Araki J. Zhao C. Ito K. (2005). Efficient Production of Polyrotaxanes from α-Cyclodextrin and Poly(ethylene Glycol). Macromolecules 38, 7524–7527. 10.1021/ma050290+

[B6] Benedini S. Zheng Y. Nitti A. Mazza M. M. A. Dondi D. Raymo F. x. i. M. (2021). Large Polarization of Push–Pull “Cruciforms” via Coordination with Lanthanide Ions. New J. Chem. 46, 221–227. 10.1039/D1NJ04358G

[B7] Bin Imran A. Esaki K. Gotoh H. Seki T. Ito K. Sakai Y. (2014). Extremely Stretchable Thermosensitive Hydrogels by Introducing Slide-Ring Polyrotaxane Cross-Linkers and Ionic Groups into the Polymer Network. Nat. Commun. 5, 5124. 10.1038/ncomms6124 25296246 PMC4214411

[B8] Chambon F. Winter H. H. (1987). Linear Viscoelasticity at the Gel Point of a Crosslinking Pdms with Imbalanced Stoichiometry. J. Rheol. 31, 683–697. 10.1122/1.549955

[B9] Cheikh W. Rózsa Z. B. Camacho López C. O. Mizsey P. Viskolcz B. Szőri M. (2019). Urethane Formation with an Excess of Isocyanate or Alcohol: Experimental and ab initio Study. Polymers 11, 1543. 10.3390/polym11101543 PMC6835639 31546721

[B10] Cho Y. Kim J. Elabd A. Choi S. Park K. Kwon T.-w. (2019). A Pyrene–Poly (Acrylic Acid)–Polyrotaxane Supramolecular Binder Network for High-Performance Silicon Negative Electrodes. Adv. Mater. 31, 1905048. 10.1002/adma.201905048 31693231

[B11] Choi S. Kwon T.-w. Coskun A. Choi J. W. (2017). Highly Elastic Binders Integrating Polyrotaxanes for Silicon Microparticle Anodes in Lithium Ion Batteries. Science 357, 279–283. 10.1126/science.aal4373 28729506

[B12] Dikshit K. Bruns C. J. (2021). Post-Synthesis Modification of Slide-Ring Gels for Thermal and Mechanical Reconfiguration. Soft Matter 17, 5248–5257. 10.1039/d0sm02260h 33949424

[B13] Fleury G. Schlatter G. Brochon C. Travelet C. Lapp A. Lindner P. (2007). Topological Polymer Networks with Sliding Cross-Link Points: the “Sliding Gels”. Relationship between Their Molecular Structure and the Viscoelastic as Well as the Swelling Properties. Macromolecules 40, 535–543. 10.1021/ma0605043

[B14] Giraldo J. Vivas N. M. Vila E. Badia A. (2002). Assessing the (A) Symmetry of Concentration-Effect Curves: Empirical versus Mechanistic Models. Pharmacol. Ther. 95, 21–45. 10.1016/s0163-7258(02)00223-1 12163126

[B15] Gorteau V. Bollot G. Mareda J. Pasini D. Tran D.-H. Lazar A. N. (2005). Synthetic Multifunctional Pores that Open and Close in Response to Chemical Stimulation. Bioorg. Med. Chem. 13, 5171–5180. 10.1016/j.bmc.2005.05.022 15951187

[B16] Halley P. J. Mackay M. E. (1996). Chemorheology of Thermosets—An Overview. Polym. Eng. Sci. 36, 593–609. 10.1002/pen.10447

[B17] Harada A. Li J. Kamachi M. (1992). The Molecular Necklace: A Rotaxane Containing Many Threaded *α*-Cyclodextrins. Nature 356, 325–327. 10.1038/356325a0

[B18] Hart L. F. Hertzog J. E. Rauscher P. M. Rawe B. W. Tranquilli M. M. Rowan S. J. (2021). Material Properties and Applications of Mechanically Interlocked Polymers. Nat. Rev. Mater. 6, 508–530. 10.1038/s41578-021-00278-z

[B19] Hyodo K. Arisaka Y. Yamaguchi S. Yoda T. Yui N. (2019). Stimulation of Microvascular Networks on Sulfonated Polyrotaxane Surfaces with Immobilized Vascular Endothelial Growth Factor. Macromol. Biosci. 19, 1800346. 10.1002/mabi.201800346 30624848

[B20] Iida M. Goto T. Mayumi K. Maeda R. Hatakeyama K. Ito T. (2021). Fabrication of Polyrotaxane and Graphene Nanoplate Composites with High Thermal Conductivities. Polym. Compos. 42, 5556–5563. 10.1002/pc.26246

[B21] Ito K. (2007). Novel Cross-Linking Concept of Polymer Network: Synthesis, Structure, and Properties of Slide-Ring Gels with Freely Movable Junctions. Polymer 39, 489–499. 10.1295/polymj.pj2006239

[B22] Jiang L. Liu C. Mayumi K. Kato K. Yokoyama H. Ito K. (2018). Highly Stretchable and Instantly Recoverable Slide-Ring Gels Consisting of Enzymatically Synthesized Polyrotaxane with Low Host Coverage. Chem. Mater. 30, 5013–5019. 10.1021/acs.chemmater.8b01208

[B23] Junior E. A. P. Davila J. L. d’Avila M. A. (2019). Rheological Studies on Nanocrystalline Cellulose/alginate Suspensions. J. Mol. Liq. 277, 418–423. 10.1016/j.molliq.2018.12.091

[B24] Kang T. W. Tamura A. Arisaka Y. Yui N. (2021). Visible Light-Degradable Supramolecular Gels Comprising Cross-Linked Polyrotaxanes Capped by Trithiocarbonate Groups. Polym. Chem. 12, 3794–3805. 10.1039/d1py00569c

[B25] Karino T. Shibayama M. Ito K. (2006). Slide-ring Gel: Topological Gel with Freely Movable Cross-Links. Phys. B Condens. Matter 385, 692–696. 10.1016/j.physb.2006.05.293

[B26] Kato K. Yasuda T. Ito K. (2013). Viscoelastic Properties of Slide-Ring Gels Reflecting Sliding Dynamics of Partial Chains and Entropy of Ring Components. Macromolecules 46, 310–316. 10.1021/ma3021135

[B27] Kato K. Karube K. Nakamura N. Ito K. (2015a). The Effect of Ring Size on the Mechanical Relaxation Dynamics of Polyrotaxane Gels. Polym. Chem. 6, 2241–2248. 10.1039/c4py01644k

[B28] Kato K. Okabe Y. Okazumi Y. Ito K. (2015b). A Significant Impact of Host-Guest Stoichiometry on the Extensibility of Polyrotaxane Gels. Chem. Commun. 51, 16180–16183. 10.1039/c5cc07122d 26399995

[B29] Kato K. Ikeda Y. Ito K. (2019). Direct Determination of Cross-Link Density and its Correlation with the Elastic Modulus of a Gel with Slidable Cross-Links. ACS Macro Lett. 8, 700–704. 10.1021/acsmacrolett.9b00238 35619527

[B30] Kodavaty J. Venkat G. Deshpande A. P. Gummadi S. N. (2020). Molecular Association and Gelling Characteristics of Curdlan. Curr. Sci. 118, 1436–1442. 10.18520/cs/v118/i9/1436-1442

[B31] Lin Y.-C. Ito K. Yokoyama H. (2018). Solid Polymer Electrolyte Based on Crosslinked Polyrotaxane. Polymer 136, 121–127. 10.1016/j.polymer.2017.12.046

[B32] Liu C. Kadono H. Mayumi K. Kato K. Yokoyama H. Ito K. (2017). Unusual Fracture Behavior of Slide-Ring Gels with Movable Cross-Links. ACS Macro Lett. 6, 1409–1413. 10.1021/acsmacrolett.7b00729 35650803

[B33] Liu C. Kadono H. Yokoyama H. Mayumi K. Ito K. (2019). Crack Propagation Resistance of Slide-Ring Gels. Polymer 181, 121782. 10.1016/j.polymer.2019.121782

[B34] Liu C. Morimoto N. Jiang L. Kawahara S. Noritomi T. Yokoyama H. (2021). Tough Hydrogels with Rapid Self-Reinforcement. Science 372, 1078–1081. 10.1126/science.aaz6694 34083486

[B35] Matsunaga S. Tamura A. Fushimi M. Santa H. Arisaka Y. Nikaido T. (2020). Light-embrittled Dental Resin Cements Containing Photodegradable Polyrotaxane Cross-Linkers for Attenuating Debonding Strength. ACS Appl. Polym. Mater. 2, 5756–5766. 10.1021/acsapm.0c01024

[B36] Mayumi K. Ito K. (2010). Structure and Dynamics of Polyrotaxane and Slide-Ring Materials. Polymer 51, 959–967. 10.1016/j.polymer.2009.12.019

[B37] Mayumi K. Ito K. Kato K. (2015). Polyrotaxane and Slide-Ring Materials. Cambridge: Royal Society of Chemistry.

[B38] Minato K. Mayumi K. Maeda R. Kato K. Yokoyama H. Ito K. (2017). Mechanical Properties of Supramolecular Elastomers Prepared from Polymer-Grafted Polyrotaxane. Polymer 128, 386–391. 10.1016/j.polymer.2017.02.090

[B39] Noda Y. Hayashi Y. Ito K. (2014). From Topological Gels to Slide-Ring Materials. J. Appl. Polym. Sci. 131, 40509. 10.1002/app.40509

[B40] Ohmori K. Bin Imran A. Seki T. Liu C. Mayumi K. Ito K. (2016). Molecular Weight Dependency of Polyrotaxane-Cross-Linked Polymer Gel Extensibility. Chem. Commun. 52, 13757–13759. 10.1039/c6cc07641f 27797388

[B41] Okumura Y. Ito K. (2001). The Polyrotaxane Gel: A Topological Gel by Figure-Of-Eight Cross-Links. Adv. Mater. 13, 485–487. 10.1002/1521-4095(200104)13:7<485:aid-adma485>3.0.co;2-t

[B42] Rajendran A. K. Arisaka Y. Iseki S. Yui N. (2019). Sulfonated Polyrotaxane Surfaces with Basic Fibroblast Growth Factor Alter the Osteogenic Potential of Human Mesenchymal Stem Cells in Short-Term Culture. ACS Biomater. Sci. Eng. 5, 5652–5659. 10.1021/acsbiomaterials.8b01343 33405696

[B43] Samitsu S. Araki J. Kataoka T. Ito K. (2006). New Solvent for Polyrotaxane. II. Dissolution Behavior of Polyrotaxane in Ionic Liquids and Preparation of Ionic Liquid-Containing Slide-Ring Gels. J. Polym. Sci. B Polym. Phys. 44, 1985–1994. 10.1002/polb.20849

[B44] Seo J. Moon S. W. Kang H. Choi B.-H. Seo J. H. (2019). Foldable and Extremely Scratch-Resistant Hard Coating Materials from Molecular Necklace-like Cross-Linkers. ACS Appl. Mater. Interf. 11, 1–12. 10.1021/acsami.9b05738 31241308

[B45] Seo J. Hur J. Kim M.-S. Lee T.-G. Seo S. J. Han S. H. (2021). All-organic Piezoelectric Elastomer Formed through the Optimal Cross-Linking of Semi-crystalline Polyrotaxanes. Chem. Eng. J. 426, 130792. 10.1016/j.cej.2021.130792

[B46] Shinohara Y. Kayashima K. Okumura Y. Zhao C. Ito K. Amemiya Y. (2006). Small-angle X-Ray Scattering Study of the Pulley Effect of Slide-Ring Gels. Macromolecules 39, 7386–7391. 10.1021/ma061037s

[B47] Subramani R. Izquierdo-Alvarez A. Bhattacharya P. Meerts M. Moldenaers P. Ramon H. (2020). The Influence of Swelling on Elastic Properties of Polyacrylamide Hydrogels. Front. Mater. 7, 212. 10.3389/fmats.2020.00212

[B48] Sun Han Chang R. Lee J. C.-W. Pedron S. Harley B. A. Rogers S. A. (2019). Rheological Analysis of the Gelation Kinetics of an Enzyme Cross-Linked Peg Hydrogel. Biomacromolecules 20, 2198–2206. 10.1021/acs.biomac.9b00116 31046247 PMC6765384

[B49] Travelet C. Schlatter G. Hebraud P. Brochon C. Lapp A. Hadziioannou G. (2009). Formation and Self-Organization Kinetics of *α*-cd/peo-Based Pseudo-Polyrotaxanes in Water. a Specific Behavior at 30 c. Langmuir 25, 8723–8734. 10.1021/la900070v 19301842

[B50] Travelet C. Hebraud P. Perry C. Brochon C. Hadziioannou G. Lapp A. (2010a). Temperature-Dependent Structure of *α*-cd/peo-based Polyrotaxanes in Concentrated Solution in Dmso: Kinetics and Multiblock Copolymer Behavior. Macromolecules 43, 1915–1921. 10.1021/ma902686p

[B51] Travelet C. Schlatter G. Hébraud P. Brochon C. Anokhin D. V. Ivanov D. A. (2010b). Physical Gels Based on Polyrotaxanes: Kinetics of the Gelation, and Relative Contributions of *α*-cyclodextrin and Poly (Ethylene Oxide) to the Gel Cohesion. Macromol. Symp. 291, 202–211. 10.1002/masy.201050524

[B52] Vlassopoulos D. Chira I. Loppinet B. McGrail P. T. (1998). Gelation Kinetics in Elastomer/Thermoset Polymer Blends. Rheol. acta 37, 614–623. 10.1007/s003970050148

[B53] Vo N. T. Huang L. Lemos H. Mellor A. Novakovic K. (2020). Poly (Ethylene Glycol)-Interpenetrated Genipin-Crosslinked Chitosan Hydrogels: Structure, Ph Responsiveness, Gelation Kinetics, and Rheology. J. Appl. Polym. Sci. 137, 49259. 10.1002/app.49259

[B54] Yang M. Wang D. Sun N. Chen C. Zhao X. (2015). Rheological Behaviour and Cure Kinetic Studies of a Trifunctional Phenylethynyl-Terminated Imide Oligomer. High. Perform. Polym. 27, 449–457. 10.1177/0954008314555521

[B55] Yoo D.-J. Elabd A. Choi S. Cho Y. Kim J. Lee S. J. (2019). Highly Elastic Polyrotaxane Binders for Mechanically Stable Lithium Hosts in Lithium-Metal Batteries. Adv. Mater. 31, 1901645. 10.1002/adma.201901645 31148271

