# Supplementary material for: Chemorheological Monitoring of Cross-Linking in Slide-ring Gels Derived From α-cyclodextrin Polyrotaxanes
Source: Front Chem. 2022 Jul 14;10:923775. doi: 10.3389/fchem.2022.923775 (PMC9344045; doi:10.3389/fchem.2022.923775)
Supplement: Supplementary file 1 [file DataSheet1.PDF]

Supplementary Material

1 EXPERIMENTAL SECTION

1.1 Materials

35 kDa molecular weight (MW) poly(ethylene glycol) [PEG35k] (EMD Millipore Corporation), (2,2,6,6-tetramethylpiperidin-1-yl)oxyl [TEMPO] (Alfa Aesar), 1-adamantanamine hydrochloride [$AdNH_2 \cdot HCl$] (TCI Chemicals, Japan), α-cyclodextrin [α-CD], (benzotriazol-1-yloxytris(dimethylamino)phosphonium hexafluorophosphate) [BOP] reagent (Chem Impex International, Inc.), ethylene diisopropylamine [EDIPA] (Alfa Aesar), sodium hypochlorite [NaOCl] solution with 5% free chlorine (Spectrum Chemicals, USA), sodium bromide [NaBr] (Acros Organics), hydrochloric acid [HCl] (37%, Sigma-Aldrich), sodium hydroxide [NaOH], dichloromethane [DCM], dimethylformamide [DMF], dimethyl sulfoxide [DMSO] (Fisher Chemicals, USA), ethanol [EtOH] and methanol [MeOH] (Decon Laboratories, Inc.), hexamethylene diisocyanate [HMDI] and dibutyltin dilaurate [DBTDL] (TCI America) were used as received. Reverse osmosis (RO)-purified water was obtained from a central campus facility through a tap.

1.2 Synthetic Procedures

Adamantane (Ad)-capped unmodified polyrotaxane (PR) was synthesized from poly(ethylene glycol) dicarboxylate (PEGDC), α-CD, and adamantamine [$Ad\text{-}NH_2$] using the following protocol.

1.2.1 PEGDC

PEG35k (10g, 0.3 mmol) was dissolved in RO water (100 mL) maintained at pH 10 with 1 M NaOH solution (100 μL). TEMPO (100 mg, 0.6 mmol), NaBr (100 mg, 1 mmol), and NaOCl solution (15 mL) were added and the reaction was stirred at room temperature (RT) for 20 min. EtOH/MeOH, equal in amount to NaOCl solution, was added to quench any unreacted NaOCl, followed by dropwise addition of HCl (0.003 M) until the pH was 2, in order to ensure protonation of PEGDC. The polymer was extracted from the aqueous solution into DCM (over 3 aliquots of 100 mL each), dried in a rotary evaporator, and residue was dissolved in hot EtOH (100 mL) followed by overnight refrigeration to precipitate PEGDC. The product was collected by vacuum filtration and dried under vacuum at 60 °C to yield PEGDC (8 g, 80%) as a white powder, which was used without further purification.

1.2.2 Polyrotaxane (PR)

PEGDC (3g, 0.09 mmol) was dissolved in RO water (100 mL) and maintained at 80 °C with stirring. α-CD (12g, 12 mmol) was added and the solution was stirred for 30 min until it was no longer turbid. The solution was placed in a refrigerator at 4 °C overnight to precipitate the PEG$\subset$ (α-CD)$_n$ inclusion complex, or pseudo-polyrotaxane (pseudoPR), which was isolated as a white powder by lyophilization and used without further purification. The crude pseudoPR ($\sim$13 g) was dispersed in anhydrous DMF (100 mL). BOP reagent (0.48g, 1.1 mmol), $AdNH_2$ (1.6g, 1.1 mmol) – obtained from [$AdNH_2 \cdot HCl$] by washing with aqueous NaOH, extraction in DCM, and drying by rotary evaporation – and EDIPA (200 μl, 1.1 mmol) were added to the slurry and the mixture was stirred at RT for 30 min. The slurry was placed in a refrigerator at 4 °C overnight to stopper the pseudoPR, affording the crude polyrotaxane (PR). The resulting polymer was purified by multiple steps of centrifugation, first with water, then with methanol. The product was dried under vacuum at 70 °C overnight. The residue was dissolved in DMSO at a concentration

of 10% w/v, and the centrifugation and drying procedure was repeated a second time to obtain PR as a white solid (5.3g, 39%).

1.2.3 Quenching Procedure

Gel quenching experiments were performed with 1-ml samples of the $P_{10}C_5T_{25}$ pregel solution in 20 mL glass scintillation vials. The 1-ml pre-gel solutions were placed simultaneously in three separate vials, which were capped and incubated on the benchtop until MeOH (19 ml) was added to quench one reaction each at time points of 10 h, 14 h, and 18 h. Over the course of 10 minutes, the de-swelling of the quenched gels was sufficient to separate them from the walls of the scintillation vials, at which time the gels were transferred carefully by hand into sealed 8-oz glass containers containing $\sim$100 ml of MeOH. The gels were agitated in these MeOH baths overnight on an orbital shaker at 80 rpm. The MeOH was decanted and the washed gels were dried in a vacuum oven overnight at 70 °C. After recording their mass, the dried gels were re-swollen in DMSO (40 ml) for 25 h, weighed again (to calculate swelling ratios, Table S1), and cut into 25-mm discs to be mounted on the rheometer for mechanical testing.

1.3 Characterization

Nuclear Magnetic Resonance (NMR) spectroscopy was performed on a Bruker Avance-III 300 MHz NMR spectrometer. The ^{1}H NMR spectrum of PR was used to estimate an inclusion ratio of $\sim$30% (corresponding to approximately 120 α-CD rings per chain) by comparative signal integration of the Ad and α-CD resonances (Fig S1). The molecular weight (MW) of PR is therefore estimated by ^{1}H NMR spectroscopy to be $\sim$152 kDa.

1.4 Rheometry

Rheometry of pregel solutions was carried out using a shear rheometer (Anton Paar MCR 301) equipped with a 25mm parallel plate geometry and an accompanying peltier plate for temperature control, which serves as the bottom plate. Dynamic oscillatory shear experiments were used to track gelation over 18 hours at varying PR concentrations (Fig. S3), crosslinker concentration (Fig. S4) and different temperatures (Fig. S5). The viscoelasticity of the gels was measured using a gap maintained at 0.5 mm. Mechanical spectra were recorded with time at a frequency of 1 rad/s, at a strain of 1%. A layer of heavy mineral oil was placed around the sample to avoid solvent evaporation and prevent recording of false data. A time sweep on a PR solution in DMSO, devoid of catalyst and cross-linker, was carried out with and without oil present around the sample. Figure S6 shows an increase in moduli values when time sweep was carried out without oil around the sample. When oil is placed around the sample no false “gelation” is observed in the sample confirming that oil around the sample is a simple yet effective strategy to avoid solvent evaporation or pseudo-gelation.

Equation (2) in the main text is obtained by combining the following two equations, which give the slope (P) at gelation halftime and $\dot{n}_\theta$ as a function of P, respectively.

$$P = \frac{\alpha G'_\infty}{4\theta} \tag{S1}$$

$$\dot{n}_\theta = \frac{P}{RT} \tag{S2}$$

2 SUPPLEMENTAL FIGURES

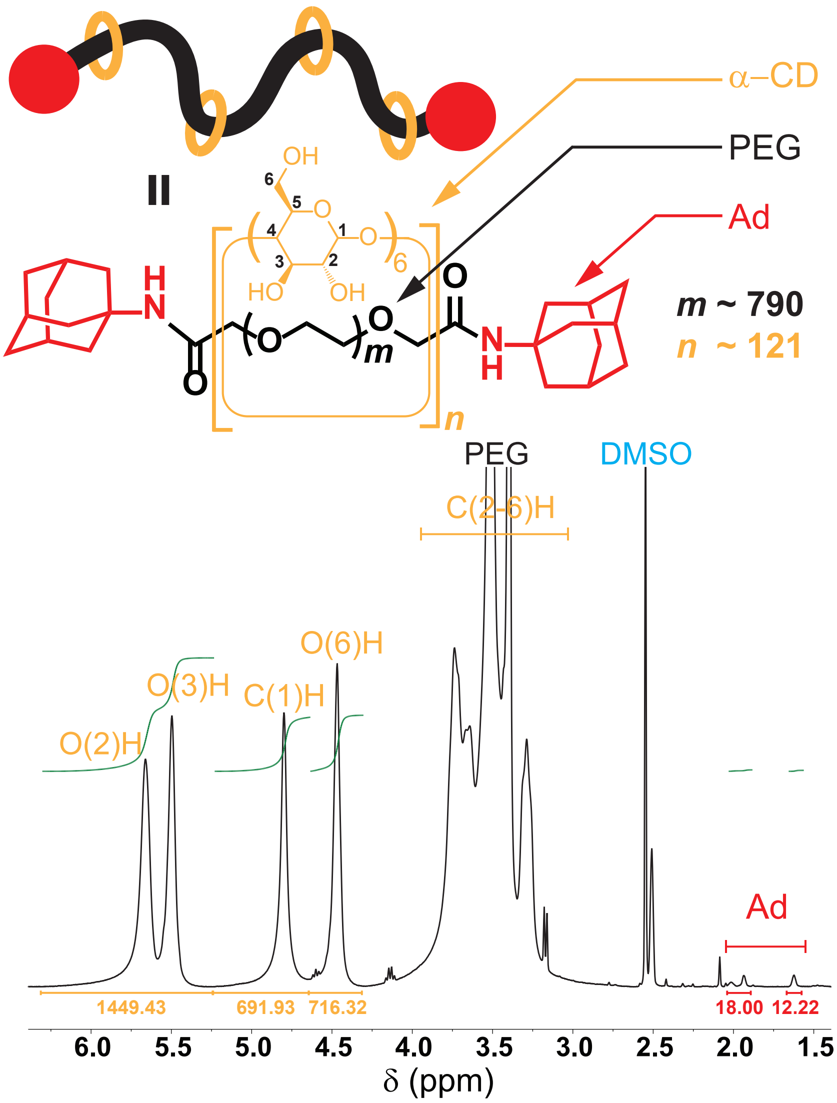

Figure S1. ^{1}H NMR spectrum (300 MHz, DMSO-d_6, 293 K) of PR.

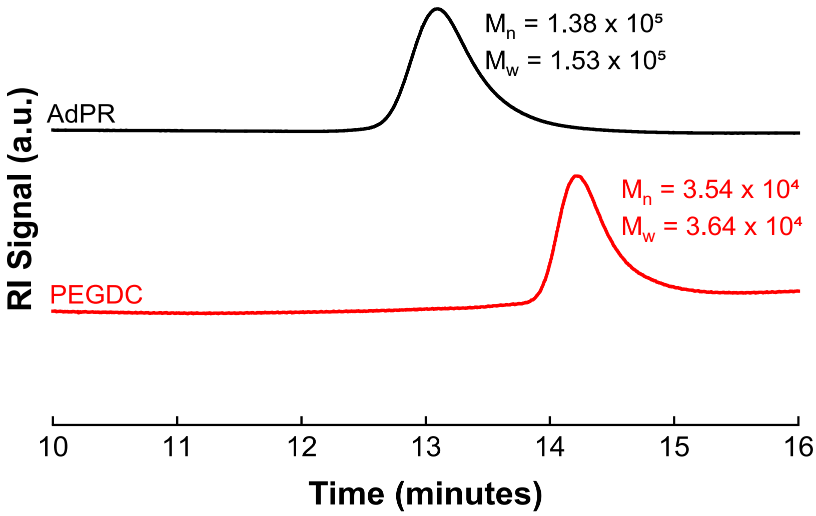

Figure S2. Determination of PR molecular weight by Gel Permeation Chromatography (GPC). DMSO was employed as the eluent with 0.2 % w/v solution of PR in DMSO. The GPC was calibrated using standard PEG molecular weights to create calibration curves.

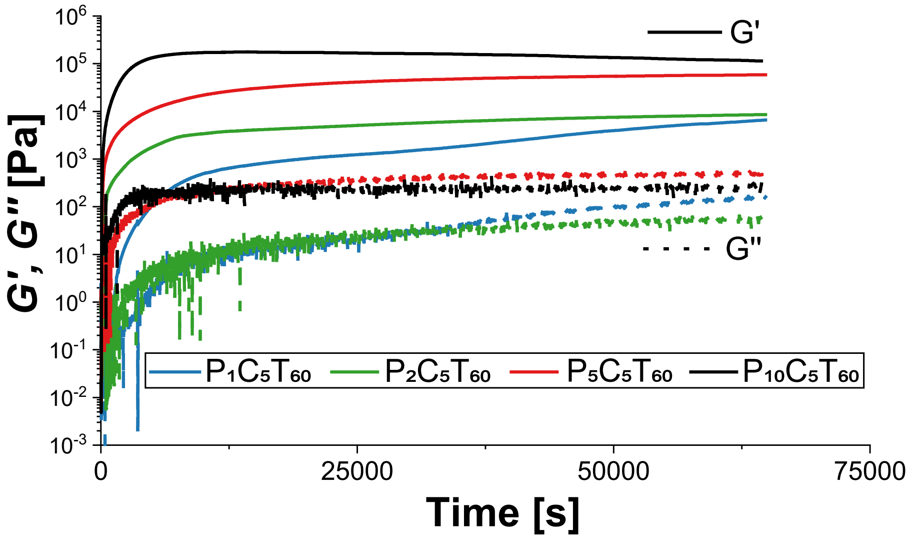

Figure S3. Rheological time sweep data of gelation specimens with varying PR concentration (1% - 10% w/v) at a constant HMDI concentration of 5 % v/v and a temperature of 60 °C

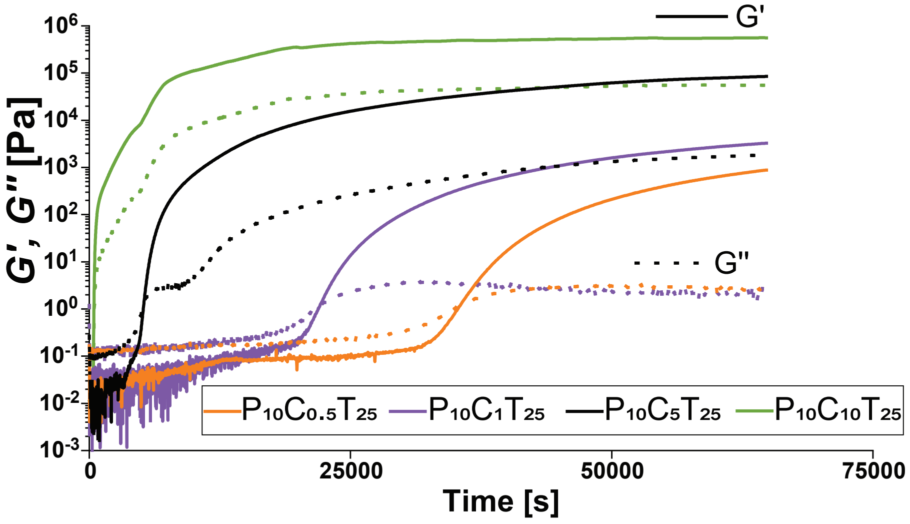

Figure S4. Rheological time sweep data of gelation specimens with varying HMDI concentration (0.5 - 10 % v/v) at a constant temperature of 25 °C.

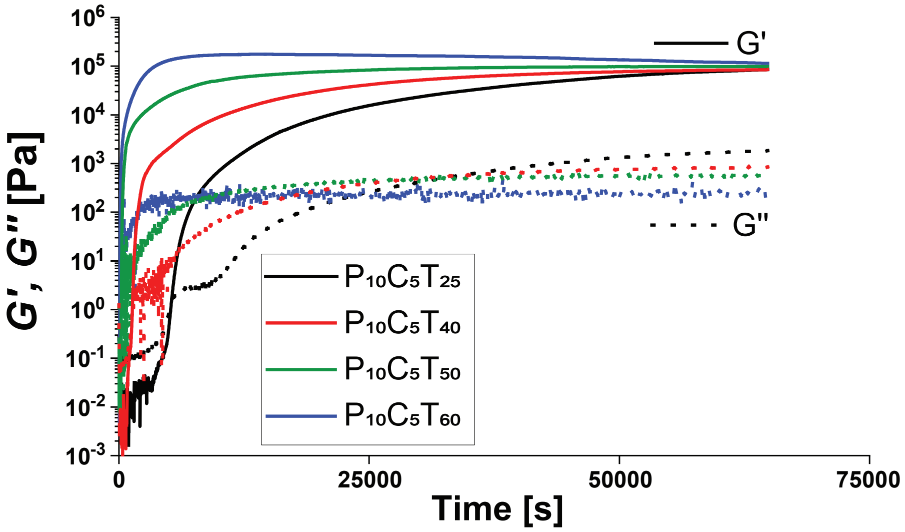

Figure S5. Rheological time sweep data of gelation specimens with varying temperature (25 - 60 °C) at a constant HMDI concentration of 5 % v/v.

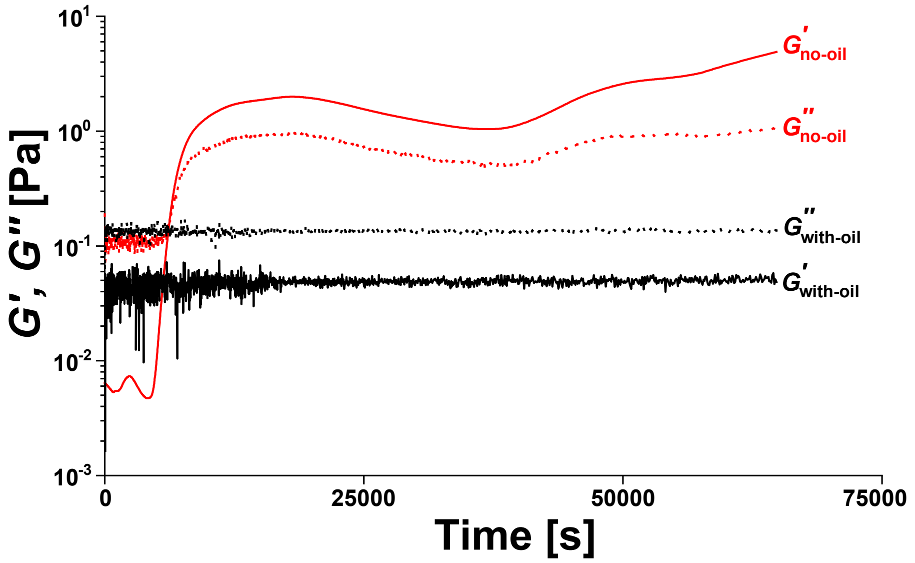

Figure S6. Rheological data for oil check. The red plot, which shows a crossover regime and increase in G' and G" is for PR solution with no oil around the sample, whereas no changes are seen for a PR solution with oil around the sample.

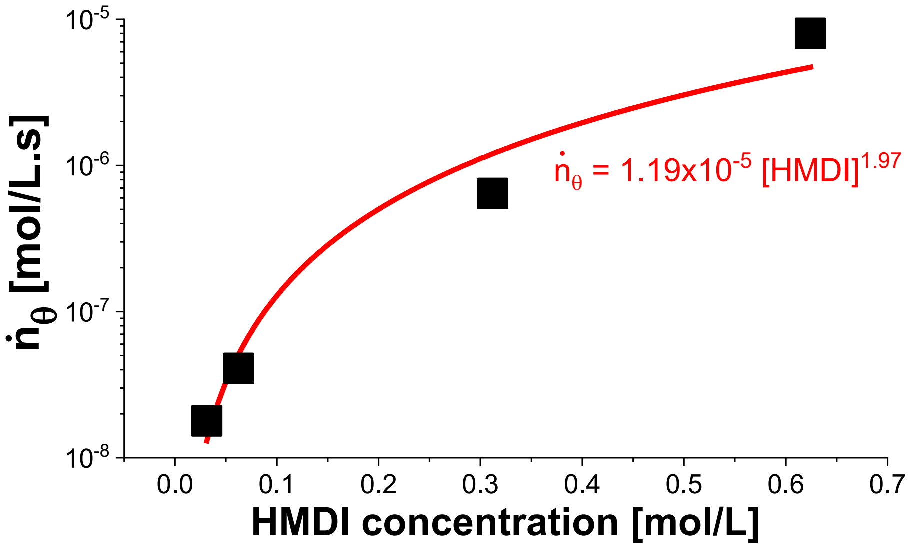

Figure S7. Dependence of HMDI concentration on kinetics. Rate of production of elastically effective cross-links showed a second order dependence on cross-linker (HMDI) concentration expressed in mol/L.

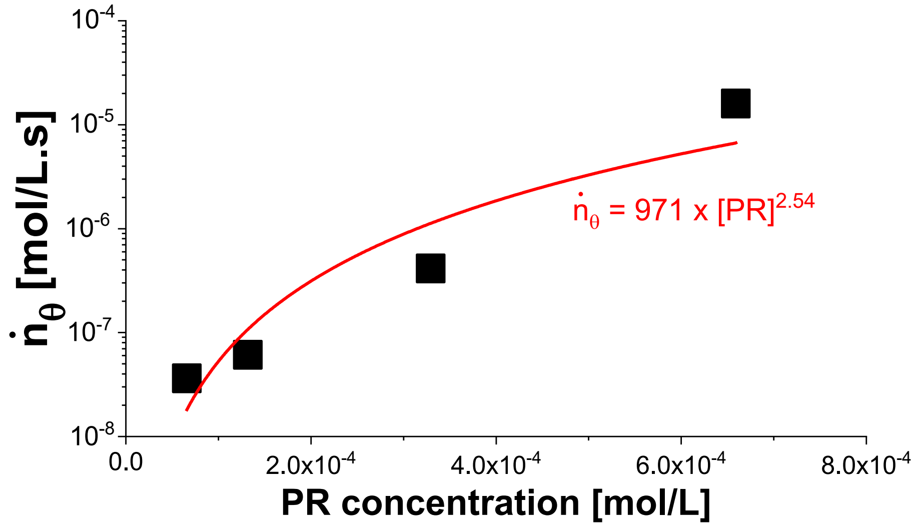

Figure S8. Dependence of PR concentration on kinetics. Rate of production of elastically effective cross-links showed a higher that second order dependence on polyrotaxane (PR) concentration expressed in mol/L.

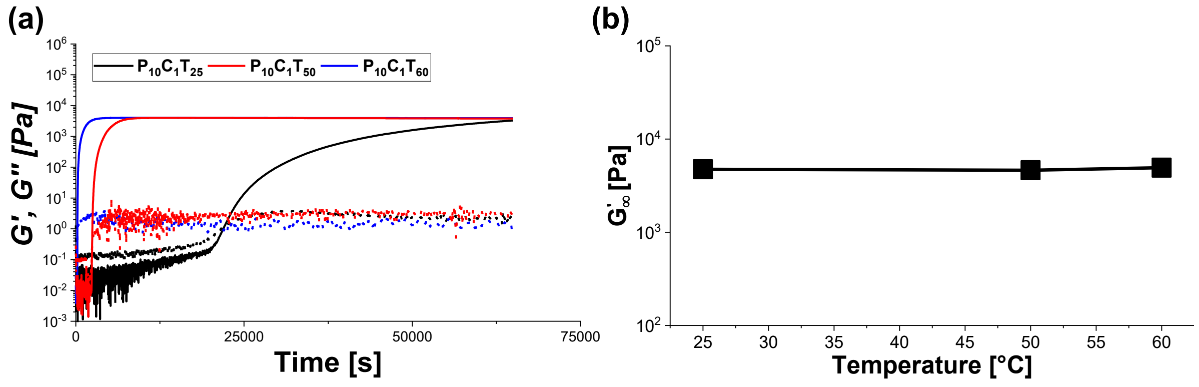

Figure S9. Temperature insensitivity of G'_∞ is also observed for $P_{10}C_1T_x$ samples

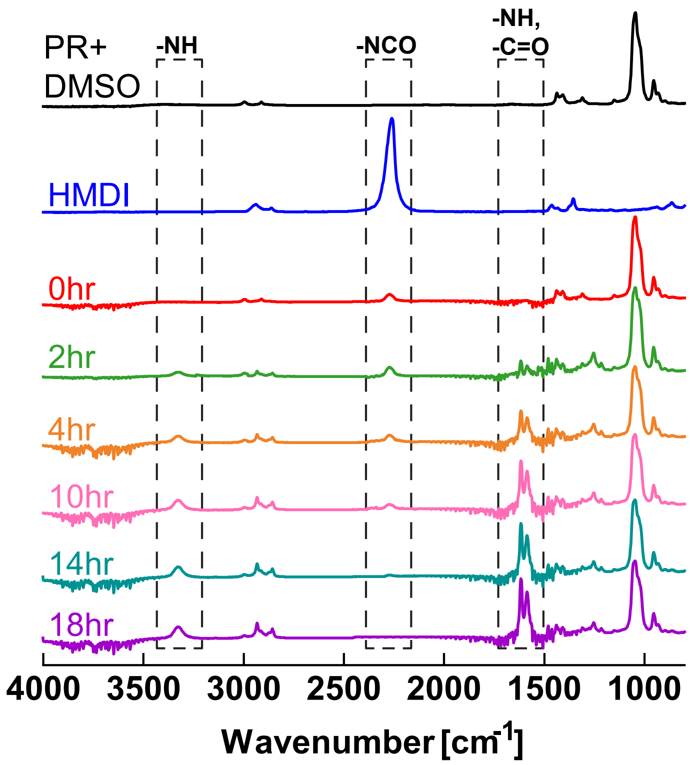

Figure S10. Fourier Transform Infrared (FTIR) spectroscopy used to track the consumption of isocyanate and formation of urethane throughout the gelation process.

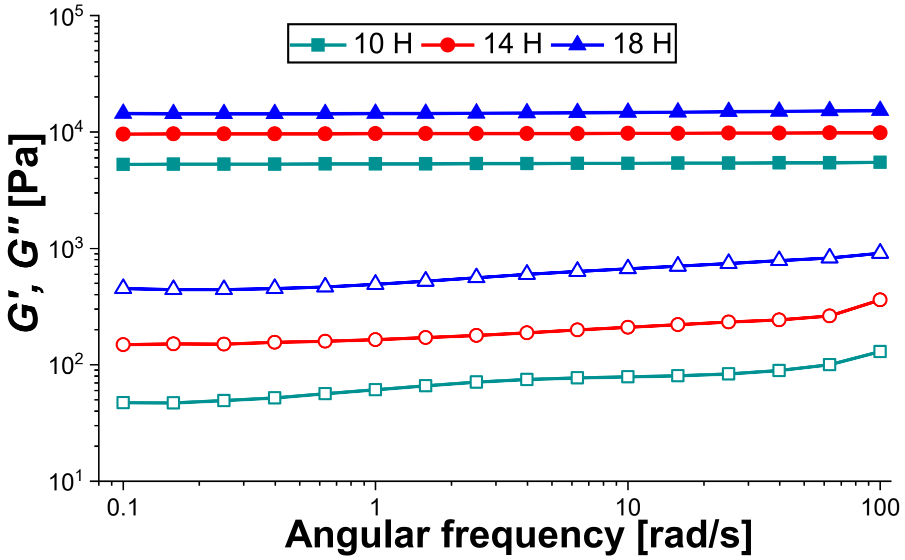

Figure S11. Frequency sweep of gels terminated at 10 hours, 14 hours, and 18 hours using methanol as the quench medium. Gels were swollen in DMSO for 24 h before testing. A strain of 1% was used for the frequency sweep between 0.1 – 100 rad/s

Table S1. Degree of swelling of $P_{10}C_5T_{25}$ quenched at different time points.

Time of gelation	Swelling ratio (by weight)
10 hours	16
14 hours	10
18 hours	8
